# Bone strain index reproducibility and soft tissue thickness influence: a dual x-ray photon absorptiometry phantom study

**DOI:** 10.1186/s41747-019-0110-9

**Published:** 2019-08-14

**Authors:** C. Messina, L. P. Piodi, L. Rinaudo, I. Emili, F. Porro, C. Buonomenna, L. M. Sconfienza, L. Vergani, F. M. Ulivieri

**Affiliations:** 1 IRCCS Istituto Ortopedico Galeazzi, Via Riccardo Galeazzi 4, 20161 Milano, Italy; 2 Dipartimento di Scienze Biomediche per la Salute, Università degli Studi di Milano, Via Pascal, 36, 20100 Milano, Italy; 3 Former: Fondazione IRCCS Ca’ Granda Ospedale Maggiore Policlinico, Gastroenterology and Digestive Endoscopy Unit, Via Francesco Sforza 35, 20122 Milano, Italy; 4 TECHNOLOGIC Srl, Lungo Dora Voghera 34/36A, 10153 Torino, Italy; 5 Postgraduate School of Radiodiagnostic, University of Milan, Via Festa del Perdono 7, 20122 Milano, Italy; 6 Postgraduate School of Physical Medicine and Rehabilitation Therapy, University of Milan, Via Festa del Perdono 7, 20122 Milano, Italy; 7 Department of Mechanical Engineering Politecnico di Milano, Via Giuseppe La Masa 1, 20156 Milano, Italy; 8 Fondazione IRCCS Ca’ Granda Ospedale Maggiore Policlinico, Nuclear Medicine, Bone Metabolic Unit, Via Francesco Sforza 35, 20122 Milano, Italy

**Keywords:** Absorptiometry (photon), Bone density, Bone strain index, Fractures (bone), Reproducibility of results

## Abstract

**Background:**

Bone strain index **(**BSI) is a tool measuring bone strain, derived from dual x-ray photon absorptiometry. It is able to characterise an aspect of bone quality that, joined to the quantity and quality parameters of bone mineral density (BMD) and trabecular bone score (TBS), permits an accurate definition of fracture risk. As no data are available about BSI precision, our aim was to assess its *in vitro* reproducibility.

**Methods:**

A Hologic spine phantom was used to perform BSI scans with three different scan modes: fast array (FA), array (A), and high definition (HD). Different soft tissue thicknesses (1, 3, 6 cm) of fresh pork rind layers as a surrogate of abdominal fat were interposed. For each scan mode, the phantom was consecutively scanned 25 times without repositioning.

**Results:**

In all scan modes (FA, A, HD) and at every fat thickness, BSI reproducibility was lower than that of BMD. The highest reproducibility was found using HD-mode with 1 cm of pork rind and the lowest one using HD-mode with 6 cm of pork rind. Increasing fat thickness, BSI reproducibility tended to decrease. BSI least significant change appeared to be about three times that of BMD in all modalities and fat thicknesses. Without pork rind superimposition and with 1-cm fat layer, BSI reproducibility was highest with HD-mode; with 3 or 6 cm fat thickness, it was higher with A-mode.

**Conclusions:**

BSI reproducibility was worse than that of BMD, but it is less sensitive to fat thickness increase, similarly to TBS.

## Key points

- Bone strain index (BSI) is an index of bone quality derived from dual-energy x-ray absorptiometry.
- BSI reproducibility was tested in a spine phantom study.
- BSI reproducibility was lower than that of bone mineral density (BMD) and tended to decrease with the increase of interposed swine fat tissue.
- BSI reproducibility was less sensitive to the increase of interposed fat thickness than BMD.
- BSI behaviour is similar to that of other bone quality indexes, such as trabecular bone score.

## Background

Osteoporosis is defined as a skeletal condition characterised by reduced bone strength, due to an impaired bone mass and compromised microarchitecture that cause an increased risk of non-traumatic fragility fractures [1]. Fragility fractures are important clinical events because they increase the disability and mortality related to this disease, with high social costs [1]. In addition, the presence of a fragility fracture itself predisposes to the recurrence of new fractures [1–3].

Dual x-ray photon absorptiometry (DXA) is considered the gold standard method for the diagnosis of reduced bone mass and for its follow-up [4]. Bone mineral density (BMD) is the most relevant parameter of bone load resistance and is calculated as bone mineral content on area (g/cm^2^). It is expressed by T-score and Z-score, indicating how the measured data diverge from the mean value of a normal population or from the mean value of a population of the same sex and age, respectively. According to the World Health Organization criteria, a T-score lower than -2.5 defines the cutoff for the diagnosis of osteoporosis in postmenopausal women and men over 50, while a Z-score lower than -2.0 represents the cutoff for the diagnosis of reduced bone mass in premenopausal women and men under 50 [5].

However, while BMD is clearly one of the major determinants of bone strength [6], the assessment of fracture risk based on BMD alone could lack sensitivity. In fact, many fragility fractures occur in osteopenic individuals (T score between -2.5 and -1.0), not only in subjects with osteoporosis (T score below -2.5) [7]. Other factors in addition to BMD account for bone strength and fracture risk, concurring to determine bone quality [8]. They are represented by trabecular architecture, bone geometry, and bone turnover. The last one is evaluated by biochemical assays, while the first two can be directly measured by bone biopsy. Recently, the use of DXA has been enriched with new software that are capable to provide information on bone microarchitecture and turnover, such as the trabecular bone score (TBS) and the hip structural analysis [9].

In this scenario, another DXA-derived software has been recently developed with the aim of further investigating bone quality, a tool called bone strain index (BSI) [10]. This software applies a mathematical model called finite element method (FEM) to DXA lumbar spine scans. FEM derived from quantitative computed tomography scans demonstrated to be predictive of experimental vertebra strength [11]. The BSI parameter uses the format of digital imaging and communications in medicine (DICOM) files to obtain an average strain field from FEM analysis, calculated within each lumbar vertebra. The force acting on the surface of each vertebra has been related to the height and weight of each patient. A classical mathematical matricial approach using a triangular mesh and FEM has been applied defining the stiffness matrix dependent from the local BMD. The result of this process is the distribution of the strain that is defined as the spatial deformation of every single element in which the vertebra was divided before the calculation [12]. In order to make this analysis easier in daily usage, the model described above has been completely automated integrating both the segmentation process and FEM analysis in a single software [10]. A recent analysis of the BSI software showed a good correlation between BSI and the yield strain applied to porcine vertebrae, meaning that BSI can be a predictor of elastic and plastic changes in the mechanical response of the bone. As a consequence, BSI could be applied in the prediction of the possible region of fracture within the vertebrae. When the software was applied to DXA scan of human vertebrae, a good correlation was found with T-score, in patients presenting T-score values lower than -3.0 and high values of BSI. Thus, high BSI values are associated with lower BMD values [10]. Finally, in recent clinical studies, BSI appeared to be useful to identify the osteoporotic patients’ subgroup particularly prone to fragility fractures [13], and to characterise young patients affected by secondary osteoporosis [14, 15].

Bone densitometry precision is one of the most important factors in clinical practice, especially for patient’s monitoring. Precision of DXA-related measurements such as BMD and TBS has been widely assessed [16–19]. Currently, no data are available about BSI precision. Thus, the aim of this study was to evaluate the reproducibility of BSI measurements on a phantom and to compare it with BMD reproducibility.

## Methods

This phantom study was carried out at the IRCCS Istituto Ortopedico Galeazzi of Milan, Italy, in 2017–2018. For the *in vitro* study, the Institutional Review Board approval was not requested, because no patients were involved.

A spine phantom (Model DPA/QDR-1, S/N: 13129, fan beam BMD = 0.985 g/cm^2^) provided by Hologic (Hologic Inc, Marlborough, Massachusetts, USA) for daily quality control was used. All measurements were performed using a QDR-Discovery W densitometer, software version n. 13.6.0.2 able to perform exams with three different scan modes: fast array (FA), array (A), and high definition (HD). To reproduce the human soft tissues, 5-mm layers of fresh pork rind were interposed as a surrogate of subcutaneous and visceral abdominal fat, given its high lipids’ content (80%). For each scan mode, the phantom was consecutively scanned 25 times without repositioning, with the specific purpose to test the best reproducibility for the software. The set of the three scan modes was performed without any layer of pork rind and then with layers creating an increasing thickness of 1 cm, 3 cm, and 6 cm. Thus, a total of 300 acquisitions (3 modes x 25 scans x 4 different pork rind thicknesses) were acquired. The region of interest (ROI) of the first scan was automatically set by the computer. At this stage, the operator could make image corrections if needed, for example in case of phantom ROI contour inaccuracies or intervertebral lines misplacement. After delineating the first ROI, the software automatically used it for the following acquisitions, thus avoiding any variability due to manual correction. Once DXA analysis was completed, data were obtained from the same ROI used for phantom BMD. For each scan, a standard measure of 190 cm and 80 kg was used in order to reproduce the same normal human body mass index (BMI) value of 22 kg/m^2^ for all acquisitions.

### Statistical analysis

Normality of the data was assessed using Shapiro-Wilk test. Mean ± standard deviation (SD) of BMD and BSI were calculated. Precision was obtained in accordance to the suggestions of the International Society for Clinical Densitometry official positions, and the coefficient of variation (CoV) was calculated as the ratio between standard deviation and mean. Least significant change percentage (LSC%) was calculated as 2.77 × CoV. Reproducibility was calculated as the complement to 100% of LSC% [20].

We first evaluated BMD and BSI mean values differences with increasing thickness and between the three scan modes using one-way analysis of variance (ANOVA) with Tukey post-hoc test, after verification of variance homogeneity according to Levene test. As the BSI HD subset showed variance in homogeneity, we used the Welch ANOVA with the Games-Howell post-hoc test. The comparison between BMD and BSI reproducibility at different thicknesses was performed by calculating the SD distribution of all measurements. The resulting distributions were again tested using one-way ANOVA. A *p* value lower than 0.05 was considered statistically significant.

## Results

The general overview of the results is reported in Table 1, with a direct comparison of BMD and BSI reproducibility for each modality with different thicknesses of interposed soft tissue. In general, without soft tissue superimposition, the LSC% of BSI was about three times higher than that of BMD, with the greatest difference for A-mode and FA-mode. This difference was somewhat lower at increasing thickness, with BSI LSC reaching the lowest value of 1.7% with 1 cm thickness of interposed soft tissue using HD-mode (*versus* BMD LSC = 0.7%). Overall, the reproducibility of BSI was lower compared to that of BMD.

**Table 1:** BMD and BSI mean values and their SD, LSC%, and reproducibility. Data are presented according to the specific scan mode and to the degree of soft tissue thickness

	FA (0 cm)		A (0 cm)		HD (0 cm)	
	BMD	BSI	BMD	BSI	BMD	BSI
Mean	0.987	2.168	0.987	2.121	1.005	2.077
SD	0.004	0.022	0.003	0.019	0.002	0.013
CoV	0.4%	1.0%	0.3%	0.9%	0.2%	0.6%
LSC	1.0%	2.8%	0.7%	2.5%	0.6%	1.8%
Reproducibility	99.0%	97.2%	99.3%	97.5%	99.4%	98.2%
	FA (1 cm)		A (1 cm)		HD (1 cm)	
	BMD	BSI	BMD	BSI	BMD	BSI
Mean	0.990	2.146	0.990	2.142	1.003	2.084
SD	0.003	0.018	0.004	0.023	0.003	0.012
CoV	0.3%	0.8%	0.4%	1.1%	0.3%	0.6%
LSC	0.9%	2.3%	1.2%	3.0%	0.7%	1.7%
Reproducibility	99.1%	97.7%	98.8%	97.0%	99.3%	98.3%
	FA (3 cm)		A (3 cm)		HD (3 cm)	
	BMD	BSI	BMD	BSI	BMD	BSI
Mean	0.980	2.178	0.981	2.159	1.001	2.081
SD	0.005	0.029	0.004	0.017	0.004	0.022
CoV	0.5%	1.3%	0.4%	0.8%	0.4%	1.1%
LSC	1.3%	3.7%	1.1%	2.2%	1.1%	3.0%
Reproducibility	98.7%	96.3%	98.9%	97.8%	98.9%	97.0%
	FA (6 cm)		A (6 cm)		HD (6 cm)	
	BMD	BSI	BMD	BSI	BMD	BSI
Mean	0.994	2.134	0.995	2.099	1.020	2.045
SD	0.005	0.025	0.005	0.023	0.006	0.029
CoV	0.5%	1.2%	0.5%	1.1%	0.6%	1.4%
LSC	1.4%	3.3%	1.3%	3.0%	1.6%	3.9%
Reproducibility	98.6%	96.7%	98.7%	97.0%	98.4%	96.1%

*BMD* Bone mineral density, *BSI* Bone strain index, *FA* Fast array, *A* Array, *HD* High definition, *CoV* Coefficient of variation, *SD* Standard deviation, *LSC*Least significant change percentage, *0 cm* Absence of interposed soft tissue

Table 2 shows a direct comparison between BMD and BSI reproducibility values with different thicknesses of interposed soft tissue using the three modalities. Regarding BMD, the highest reproducibility was 99.4% (0 cm soft tissue thickness, HD-mode), while the lowest was 98.4% (6 cm soft tissue thickness, HD-mode). For BMD, the increase in soft tissue thickness was associated with a decrease in reproducibility, which showed to be significant only for HD-mode (*p* < 0.001). On the other hand, the highest value of BSI reproducibility was 98.3% (1-cm soft tissue thickness, HD-mode), whereas the lowest was 96.1% (6 cm soft tissue thickness, HD-mode).

**Table 2:** Comparison between BMD and BSI reproducibility at different thicknesses

	BMD (Fast array)					BSI (Fast array)				
Pork rind thickness	0 cm	1 cm	3 cm	6 cm	*p* value	0 cm	1 cm	3 cm	6 cm	*p* value
SD	0.004	0.003	0.005	0.005	n.s.	0.022	0.018	0.029	0.025	n.s.
CoV	0.4%	0.3%	0.5%	0.5%		1.0%	0.8%	1.3%	1.2%	
LSC	1.0%	0.9%	1.3%	1.4%		2.8%	2.3%	3.7%	3.3%	
Reproducibility	99.0%	99.1%	98.7%	98.6%		97.2%	97.7%	96.3%	96.7%	
	BMD (Array)					BSI (Array)				
Pork rind thickness	0 cm	1 cm	3 cm	6 cm		0 cm	1 cm	3 cm	6 cm	
SD	0.003	0.004	0.004	0.005	n.s.	0.019	0.023	0.017	0.023	n.s.
CoV	0.3%	0.4%	0.4%	0.5%		0.9%	1.1%	0.8%	1.1%	
LSC	0.7%	1.2%	1.1%	1.3%		2.5%	3.0%	2.2%	3.0%	
Reproducibility	99.3%	98.8%	98.9%	98.7%		97.5%	97.0%	97.8%	97.0%	
	BMD (HD)					BSI (HD)				
Pork rind thickness	0 cm	1 cm	3 cm	6 cm		0 cm	1 cm	3 cm	6 cm	
SD	0.002	0.003	0.004	0.006	< 0.001	0.013	0.012	0.022	0.029	< 0.001
CoV	0.2%	0.3%	0.4%	0.6%		0.6%	0.6%	1.1%	1.4%	
LSC	0.6%	0.7%	1.1%	1.6%		1.8%	1.7%	3.0%	3.9%	
Reproducibility	99.4%	99.3%	98.9%	98.4%		98.2%	98.3%	97.0%	96.1%	

*BMD* Bone mineral density, *BSI* Bone strain index, *HD* High definition, *CoV* Coefficient of variation, *SD* Standard deviation, *LSC*Least significant change percentage, *0 cm* Absence of interposed soft tissue, *n.s.* Not statistically significant

Table 3 shows the comparisons between the mean values of BMD and BSI at 0 cm and 6 cm soft tissue thickness for each scan mode. There was a significant increase in BMD values in each scan mode, with the highest difference of 1.46% for HD-mode and the lowest difference of 0.76% for FA-mode. Differently from BMD, BSI values significantly decreased from 0 to 6 cm soft tissue thickness in all scan modes, and the variation was more pronounced than for BMD. In fact, the highest negative variation was 1.57% for FA-mode, while the lowest negative variation was 1.03% in A-mode.

**Table 3:** Comparison of BMD and BSI mean values at 0 and 6 cm of pork rind layers for each scan modality

	FA		Array		HD	
	BMD	BSI	BMD	BSI	BMD	BSI
0 cm	0.987	2.168	0.987	2.121	1.005	2.077
6 cm	0.994*	2.134*	0.995*	2.099*	1.020*	2.045*
Difference 0–6 cm	+ 0.007	-0.034	+ 0.008	-0.022	+ 0.015	-0.032
% difference 0–6 cm	+ 0.76%	-1.57%	+ 0.83%	-1.03%	+ 1.46%	-1.55%
Highest LSC% (§)	1.4% (6 cm)	3.7% (3 cm)	1.3% (6 cm)	3.0% (both 1 and 6 cm)	1.6% (6 cm)	3.9% (6 cm)

*BMD* Bone mineral density, BSI Bone strain index, *FA* Fast array, *HD* High definition, *LSC%* Least significant change percentage, *0 cm* No pork rind layers. § indicates at which soft tissue thickness there was the highest value of LSC% in each scan modality

*p < 0.05 when compared with no soft tissue superimposition (0 cm)

## Discussion

This is the first study in which the reproducibility of BSI is tested on a phantom, showing that this new software has higher CoV and LSC% compared to BMD, being about two to three times higher. As a consequence, BSI reproducibility was lower than that of BMD, and it ranged from 96.1% (6 cm soft tissue thickness, HD-mode) to 98.3% (1 cm soft tissue thickness, HD-mode). BMD reproducibility confirmed to be very good, but was affected by the increase of soft tissue thickness.

This is not the first phantom study aimed to evaluate the reproducibility of DXA measurements. The important aspect of these studies relies on the fact that phantoms are usually less affected by external factors, and this represents a methodological advantage to better understand the working principle of new techniques, such as the BSI. Previous studies have been published with data regarding BMD and TBS reproducibility [16–18, 21]. Concerning TBS, phantom studies showed that its reproducibility is somewhat lower compared to that of BMD, being between 96.4–98.3% [21] and 97.7–98.3% [18]. Usually, BMD reproducibility is known to be very good and typically represents the standard of reference for other DXA-based measurements. This was confirmed by our study, as BMD showed high values of reproducibility being around 99% in all the three scan modalities. On the other hand, the reproducibility of BSI was lower than that of BMD, and this finding has implication in clinical practice especially for patient’s monitoring. From a clinical point of view, the lower is the reproducibility the longer has to be the time of follow-up to observe a clinically significant variation.

From a practical point of view, the increase in soft tissue thickness is a condition that is frequently found in clinical DXA routine, in patients with high values of BMI and waist circumference. In our study, we tried to understand the behaviour of this new technology by simulating this frequent clinical scenario, showing that BSI, despite having lower reproducibility, was equally affected as BMD. The increase in soft tissue thickness was associated to a reduction of precision, but this was statistically significant for both BMD and BSI only for the HD-mode. Thus, we found a detrimental effect of increasing soft tissue on BSI reproducibility, similar to that of BMD.

When considering BMD and BSI mean values difference between 0 and 6 cm, we notice again a similar behaviour of these two parameters, as a statistically significant variation was found for both for BMD and BSI, despite the first increased its value and the latter showed a decrease. This divergence is expected, as higher BSI values are associated to lower BMD values and vice versa. Nevertheless, the amplitude of variation between 0 and 6 cm was slightly higher for BSI compared to BMD, a difference that was more evident for the FA-mode.

This study presents some limitations. The first one is that we evaluated BSI reproducibility on a phantom that was designed for BMD quality control. Thus, the intrinsic value of BSI measurement may not be directly transferred to clinical practice, despite the evaluation of reproducibility remains independent from BSI significance. Another limitation is that we performed this study on a single phantom with a single densitometer. Thus, these results may present variations when applied to other settings, and the precision of this software may vary when a different phantom or densitometer is used, and of course, the precision may vary among patients with different degrees of bone quality. Lastly, we simulated fat tissue using a material (pork rind) which is similar but not equal to human fat; therefore, we may not directly apply these results to real BMI variations.

In conclusion, this phantom study assessed the reproducibility of BSI compared to that of BMD in different scan modes and interposed fat thicknesses, showing that BSI reproducibility was overall lower than that of BMD and only slightly negatively influenced by interposed fat thickness increase respect to BMD.

## Abbreviations

A: Array
BMD: Bone mineral density
BSI: Bone strain index
CoV: Coefficient of variation
DXA: Dual x-ray photon absorptiometry
FA: Fast array
FEM: Finite element method
HD: High definition
LSC%: Least significant change percentage
ROI: Region of interest
SD: Standard deviation
TBS: Trabecular bone score
